# Mood Induction in Depressive Patients: A Comparative Multidimensional Approach

**DOI:** 10.1371/journal.pone.0030016

**Published:** 2012-01-09

**Authors:** Irina Falkenberg, Nils Kohn, Regina Schoepker, Ute Habel

**Affiliations:** 1 Department of Psychiatry and Psychotherapy, RWTH Aachen University, Aachen, Germany; 2 JARA Brain – Translational Brain Medicine, Jülich, Aachen, Germany; 3 Virtual Project House – Gender and Technology, RWTH Aachen University, Aachen, Germany; 4 Department of Psychiatry and Psychotherapy, University of Marburg, Marburg, Germany; 5 Section of Neuroimaging, Institute of Psychiatry, King's College London, London, United Kingdom; University of Groningen, Netherlands

## Abstract

Anhedonia, reduced positive affect and enhanced negative affect are integral characteristics of major depressive disorder (MDD). Emotion dysregulation, e.g. in terms of different emotion processing deficits, has consistently been reported. The aim of the present study was to investigate mood changes in depressive patients using a multidimensional approach for the measurement of emotional reactivity to mood induction procedures. Experimentally, mood states can be altered using various mood induction procedures. The present study aimed at validating two different positive mood induction procedures in patients with MDD and investigating which procedure is more effective and applicable in detecting dysfunctions in MDD. The first procedure relied on the presentation of happy vs. neutral faces, while the second used funny vs. neutral cartoons. Emotional reactivity was assessed in 16 depressed and 16 healthy subjects using self-report measures, measurements of electrodermal activity and standardized analyses of facial responses. Positive mood induction was successful in both procedures according to subjective ratings in patients and controls. In the cartoon condition, however, a discrepancy between reduced facial activity and concurrently enhanced autonomous reactivity was found in patients. Relying on a multidimensional assessment technique, a more comprehensive estimate of dysfunctions in emotional reactivity in MDD was available than by self-report measures alone and this was unsheathed especially by the mood induction procedure relying on cartoons. The divergent facial and autonomic responses in the presence of unaffected subjective reactivity suggest an underlying deficit in the patients' ability to express the felt arousal to funny cartoons. Our results encourage the application of both procedures in functional imaging studies for investigating the neural substrates of emotion dysregulation in MDD patients. Mood induction via cartoons appears to be superior to mood induction via faces and autobiographical material in uncovering specific emotional dysfunctions in MDD.

## Introduction

In major depressive disorder (MDD), anhedonia (the reduced ability to experience pleasure), reduced positive affect and prevailing negative affect [1], [2] characterize as main clinical symptoms the affective domain of the disorder. As MDD is a disorder of mood, when studying MDD it is important to clearly define what is meant, when talking about mood and emotion (e.g. [3]). Commonly mood may be defined as a slow-moving feeling state, that is not strongly related to environmental cues or specific stimuli, whilst emotions reflect stimulus related responses or reactions to significant stimuli (e.g. [3], [4]). Although mood and emotion can thus be distinguished, both may interact upon each other, as a negative mood state can enhance the probability for experiencing negative emotions (for a detailed discussion see [4]). The definition of MDD mostly refers to altered mood states and does not necessarily involve altered emotional reactivity. The influence of mood states on emotional reactivity and their importance in MDD (e.g. anhedonia as a core feature) form the basis of three influential theories of emotional reactivity in MDD. One view is negative potentiation, which postulates that enduring negative mood in MDD enhances emotional reactivity to negative stimuli. Positive attenuation on the other hand holds that the negative mood state leads to decreased emotional reactivity in response to positive stimuli. Both processes might occur in MDD patients simultaneously. A third view proposes emotion context insensitivity (ECI, [5]) with blunted emotional reactivity to both positive and negative stimuli due to the depressed mood state (e.g. [3]).

A recent meta-analysis on emotional reactivity in MDD [3] included 19 laboratory studies and found that emotional reactivity was decreased both in response to positive and negative stimuli. Thus results were in support for the positive attenuation hypothesis and even more for the ECI view, since ECI proposes reduced emotional reactivity to negative *and* positive stimuli. Nevertheless, Bylsma and colleagues also observed a large heterogeneity across studies which could not be sufficiently explained, but may largely be due to the underpowered samples in this meta-analysis.

Since MDD is a mood disorder, the investigation of mood changes may be regarded as a promising research approach. Investigating mood states in experimental settings can be done by experimentally altering mood with mood induction procedures. Various mood induction procedures, such as films [6], emotional verbal material [7], emotional facial expressions and specific instructions [8]–[10], odorous [11] or visual stimulation and manipulated feedback [12] have been used and described. In non-clinical samples, positive mood-induction procedures have been demonstrated to improve performance on some types of cognitive tasks, such as creative problem solving [13] and cognitive flexibility [14], while negative mood induction decreased performance [15]–[17]. In clinical samples, positive mood induction has been found to increase the number of positive future events anticipated by a group of dysphoric adolescents [18]. Furthermore, positive mood induction has also been found to reduce dysfunctional attitudes in patients with depression [19].

Mood induction procedures are manifold, relying on verbal visual and auditory material. Although mostly at least moderately successful, the variety of such mood induction procedures is not all sufficiently reliable, valid, and comparable or comprising controlled stimulus material. Therefore, comparisons of different study results are limited.

Positive mood induction has been shown to yield lower effect sizes compared to negative mood induction in various procedures, although all procedures demonstrated the ability to change the mood state effectively with medium to large effect sizes [20]. An important modulating factor of mood induction procedures is demand characteristics, e.g. whether a participant is explicitly asked to change his mood according to instructions. Mood induction procedures, which directly ask their participants to try to achieve a certain mood might overestimate the mood change due to various processes [20]. Therefore, the development of a procedure, which implicitly leads to mood change by way of emotional reactivity, might be able to control this confound.

The aim of the present study was twofold: First, we aimed to validate the applicability of two different mood induction procedures in MDD patients. Second, we aimed to assess emotional reactivity during mood induction multidimensionally in three response systems particularly defining emotional reactivity [21],[3]: (1) experiential/subjective, (2) behavioral/expressive and (3) peripheral physiological responses, namely skin conductance measures. According to this definition, emotions involve experiential, behavioural and physiological reactions, and constitute the primary motivational component of mental operations and subsequent behaviour. In this context the function of “somatic markers” [22] has been emphasized. Somatic markers contribute emotional activation to cognitive processing and have primarily been introduced into decision making using skin conductance as physiological measure of somatic marker activity. Skin conductance generally relates to arousal and reacts to novelty of a stimulus [23], [24] as well as to omission of an expected stimulus [25]. Emotional, significant or intense stimuli also commonly result in elevated skin conductance responses (SCR) [24].

The first mood induction procedure relied on the presentation of emotionally expressive faces (by use of visual stimulation and specific instructions; [10]. The effectiveness of this method has been documented extensively in clinical and non-clinical samples [10], [26], [27]. So far, however, it has not been applied to patients with MDD.

The second procedure included a newly developed and validated stimulus set of funny and neutral cartoons.

Both approaches work in different ways to achieve the elevation of a mood state. While the mood induction via visual stimulation and specific instructions relies on a slow, guided change of mood by conscious effort [10] and external stimulation, mood induction via cartoons might work by accumulation of several positive emotional reactions to the cartoons leading to a more positive mood state. This paradigm might impact on mood, as humorous material cannot only induce exhilaration, but can also have a broader impact on mood in general (for discussion of the influence of humor and laughter on mood see [28]).

Therefore we were interested in differential effectiveness of these procedures as they draw on different strategies. The first changes mood directly and intentional (internally triggered, intentional, via episodic memory), while the cartoon mood induction changes mood via presentation of positive stimuli and positive emotional reactivity. Emotional reactivity impacts on the effectiveness of mood induction, as “moods may result from the vestiges of emotion” [4] (externally triggered, unintentional, via exhilaration).

We hypothesized that (1) positive mood induction would be successful in both the patient and the control groups, but that a smaller subjective effect would be observed in the patient group, as this would be congruent with the positive attenuation and ECI views of emotional reactivity in MDD [5]. (2) We hypothesized facial expression to be diminished in patients as this is a common finding in patients with MDD in various settings [29], [30]. (3) We hypothesized greater phasic changes of skin conductance for the cartoons, as cartoons supply novel information during each presentation, which potentiate phasic reactivity [23].

We expected the two procedures to differ in their mood-enhancing effects, with the cartoon procedure evoking stronger mood changes in both patients and controls, since we propose the effect of humor relies on effective external stimuli on mood to be stronger than the internal effort to generate positive affect based on facial stimuli and imagination and memory techniques. Furthermore, undisturbed affective susceptibility to humorous material has been demonstrated in MDD [31].

Findings on peripheral physiological responses in MDD have so far been heterogeneous. Some studies report lower phasic and tonic skin conductance [32], while others report unchanged or elevated activity [33]. Hence, we assumed that the stimulus properties and nature of the task would play an import role in the pattern that would emerge for MDD patients. Positive emotional reactivity to cartoons likely leads to stronger phasic reactions compared to the more slowly responding and steadier mood changes due to the visually supported memory and imagination processes.

## Methods

### Participants

16 patients with MDD (eight males and eight females, mean age = 37, *SD* = 15) and 16 healthy controls (eight males and eight females, mean age = 35, *SD* = 14) matched for age, gender and education (patients: mean education = 11.6 years, *SD* = 2.2; controls: mean education = 11.8, *SD* = 2.5) were recruited for the study (Table 1). All participants were right handed and Caucasian. Major depression was confirmed by a structured clinical interview (German version of the Structured Clinical Interview, SCID, [34] for DSM-IV by one of the authors (NK). Patients were inpatients from the Department of Psychiatry and Psychotherapy of the RWTH Aachen University Hospital. Exclusion criteria for subjects were: substance abuse for the last six months and (other) psychiatric or neurological illness based on the SCID (Axis I). Only patients with unipolar depression were included. Anxiety disorders were excluded if the first occurrence of the disorder preceded diagnosis of depression. The study was approved by the institutional review board of the Medical Faculty, RWTH Aachen University and was in accordance with the Declaration of Helsinki (2008). A detailed description of the study was supplied to the participants, and written informed consent was obtained. All the subjects had normal or corrected to normal visual acuity. Psychopathological assessment included the Hamilton Depression Scale [35] and Beck's Depression Inventory [36].

**Table 1:** Demographic and neuropsychological characteristics for patients and controls.

	Patients	Patients (male)	Patients (female)	Controls	Controls (male)	Controls (female)	t-value Patients vs. Controls	SSRI/SNRI (n = 6)	Other medication (n = 10)	SSRI/SNRI Vs. other
**HAMD score**	14.8 (4.2)	14.1 (3.7)	15.6 (4.8)	-	-	-.	-	14.8 (3.5)	14.9 (4.8)	t_16_ = −.29; p = .98
**BDI score**	21.4 (9.4)	21.7(10.3)	21.1 (9.1)	3.2 (3.1)	2.1 (2.3)	4.4 (3.2)	**t_30_ = 6.8; p<.001**	20.6 (9.3)	21.9 (9.9)	t_16_ = .26; p = .798
**Age**	37.4 (15.2)	40.5(13.8)	34.5(13.9)	35 (14.2)	34.75 (14.2)	35.2 (15.2)	t_30_ = .468; p = .64	35.17 (17.5)	38.8 (14.5)	t_16_ = −.45: p = .66
**Education (years)**	11.6 (2.2)	11.5 (2.7)	11.7 (1.6)	11.8 (2.5)	12.1 (3.2)	11.6 (1.8)	t_30_ = −.357; p = .72	11 (1.8)	12 (2.4)	t_16_ = −.22; p = .83
**MWT-B**	105.8 (14.3)	108.8 (17.8)	103.1(10.9)	104.6 (15.3)	102.8 (16.7)	106.5 (14.6)	t_30_ = .157; p = .87	105.8 (7.5)	105.8 (17.9)	t_16_ = .85; p = .933
**RWT (items)** clustering	12.9 (3.8)	12.9 (4.6)	13.0 (3.5)	14.3 (3.9)	13.3 (3.3)	15.4 (4.4)	t_30_ = −.987; p = .33	12.7 (4.5)	13.1 (3.7)	t_16_ = −.21;p = .837
**RWT (items)** switching	13.0 (4.0)	14.0 (4.4)	12.1 (3.8)	14.1 (1.9)	13.9 (2.1)	14.4 (1.9)	t_30_ = −1.0; p = .32	13.7 (3.9)	12.6 (4.3)	t_16_ = .51; p = .618
**TMT-A (s.)**	34.4 (14.6)	34.6(14.6)	34.3 (15.5)	24.1 (8.3)	21.1 (5.3)	27.1 (9.9)	**t_30_ = .2.22; p = .03**	31.2 (14.8)	36.6 (14.9)	t_16_ = −.47; p = .65
**TMT-B (s)**	61.1(34.2)	57.3(23.3)	64.5 (43.0)	45.1 (18.1)	43.4 (17.1)	46.9 (20.2)	t_30_ = .1.48; p = .15	43.7 (9.2)	72.8 (40.2)	t_16_ = 1.49; p = .16
**WMS-R (items)**	14.1 (3.3)	15.0 (3.6)	13.3 (3.0)	14.4 (2.2)	15.5 (2.3)	13.4 (1.7)	t_30_ = −.37; p = .71	15.0 (3.8)	13.4 (3.1)	t_16_ = .89; p = .388

Subdivided by gender and for patients by medication. Significant differences are marked in bold.

Six of the patients were treated with SSRI or SNRI, one patient received a combination of SSRI and SNRI, two patients a combination of SSRI and a tetracyclic antidepressant, one patient a NDRI and an atypical neuroleptic, four patients were treated with SNRI and tetracyclic antidepressants, one patient was treated with SNRI and a tricyclic antidepressant and one patient was treated with a MAO inhibitor.

### Stimuli

#### Experimental setup

All experimental paradigms were presented using Presentation software package 10 (Neurobehavioral Systems, San Francisco, USA). A video camera positioned at the laptop recorded facial expressions during the procedures. The camera was placed in front of the participants' faces at a standardized distance of 0.8m, to allow for body movement during the procedures.

The mood induction procedures were counterbalanced to exclude transfer effects. We counterbalanced the happy and neutral mood induction and the Cartoons separately, by permuting NMI and HMI and we changed the sequence of these two mood induction procedures separately. This resulted in cells of 4 participants receiving one permutation. All participants were tested between 10 am and 4 pm.

#### Standardized Mood induction

**Figure 1:**
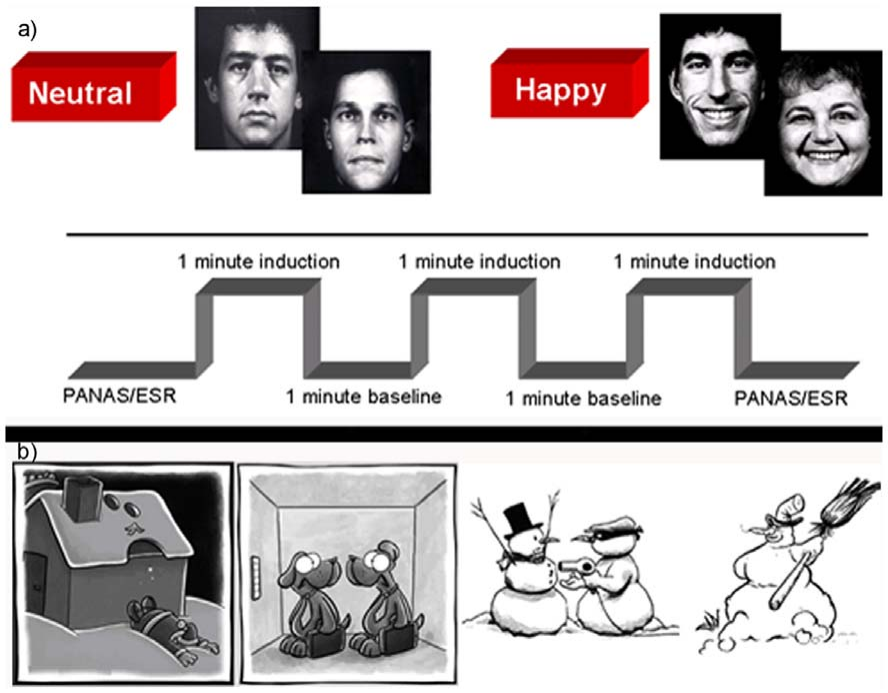
Examples of the stimulus material used in the experiment. a) Description of mood induction paradigm with sample pictures from the FEBA faces data set [e.g. 10] b) Sample cartoons from the newly developed cartoon paradigm.

The method for mood induction has been previously described in detail and used in several studies to investigate emotional processing in healthy controls and different clinical groups [10], [26], [27]. The same stimuli were applied in this study (Fig. 1a). Briefly, Caucasian professional actors were instructed to display the emotions while their pictures were taken. The models were draped in black fabric and photographed against a black backdrop to eliminate all clothing and ambient distractors. This set of photographs was reviewed by six raters for asymmetry and for ambiguity of expressed emotion. Only unitary and genuine facial expressions were retained. The method demonstrated small intraindividual variability and high retest reliability behaviorally. Presenting happy and neutral facial expressions, the task instructions were as follows: “During this task, I would like you to try to become happy [remain neutral]. To help you do that, I will be showing you slides with faces expressing happiness [neutral emotion]. Look at every face and use it to help you to feel happy [feel neutral].”

Subjects viewed the stimuli self-paced and participated in 2 conditions (happy and neutral mood induction) while videos of emotional facial expressions and skin conductance responses (SCR) were acquired. These mood induction conditions lasted for three minutes each.

Dependent measure for quantifying the subjective mood induction effect was the Positive and Negative Affect Scale (PANAS, [37]) a 5 point unipolar intensity scale, which includes 20 items for factor referenced emotional descriptors for orthogonal positive and negative dimensions. The scale required ratings of “How did you actually feel during the last few minutes?”

In this mood induction (MI) recruitment of autobiographic material was encouraged and participants were afterwards asked what they imagined to get into the mood specified. All patients and controls referred to positive autobiographical memories in order to get into a positive mood. For the neutral mood induction nearly an equal number of patients also recruited autobiographical material or imagined subjectively neutral everyday life scenes.

#### Cartoon paradigm

Cartoons were selected from various sources on the internet, and afterwards validated and standardized. We only took single picture cartoons with no more than 8 captioned words (Fig. 1b). Every picture was converted to a greyscale and standardized for brightness. Picture size was kept constant at 640x640 dpi. Captioned cartoons were changed to Arial font with a size of 12, in order to control for differences in appearance. Equivalent neutral pictures were also generated by removing the punch line or by selecting the first picture out of a multiple picture cartoon. Neutral pictures were standardized in the same manner as described above.

A detailed description of the cartoon stimuli evaluation and standardization is given in the Supplementary Material S1.

#### Cartoon stimulus presentation

In this study, each picture was shown for a fixed duration of 7s. Seven seconds were chosen, because of a mean viewing time of 5.5s plus two standard deviations (*SD* = 0.96s), to guarantee sufficient time for comprehension of the cartoon. The picture was followed by a 9-point rating scale for funniness, asking how funny the participant thought the picture was (1 = not funny at all and 9 = very funny). The rating scale also had a fixed duration of seven seconds to allow skin conductance to reach baseline level. 80 Cartoons and 80 neutral pictures were shown in a randomised order for each participant. Cartoons and neutral pictures were presented intermixed, thus a distinction between reactivity to neutral pictures and cartoons could not be distinguished in this experiment.

### Materials

#### Neuropsychological assessment

A neuropsychological test battery was used to characterize cognitive functioning in patients and controls, including tests for crystallized verbal intelligence [40], working memory [41], verbal fluency [42] and cognitive flexibility [43] (see Table 1 for details) From the neuropsychological profile only TMT-A values differed significantly between patients and controls (*F*(1,29) = 5.730; *p*<0.05; *η_p_^2^* = 0.175; *η_p_^2^* = effect size; partial eta squared; [44]).

#### Electrodermal measurement and analyses

Electrodermal activity (EDA) was assessed via a Varioport-B biosignal recorder (Becker Meditec, Karlsruhe, Germany) with a sampling rate of 16 Hz on the thenar and hypothenar eminence of the non-dominant hand with two Ag-AgCl electrodes which were applied with an electrode paste of 0.5% saline in a neutral base (Med Associates TD-246) and had a DC excitation voltage of 0.5V. The setup of our experiment and the measurement of EDA with the Varioport-B recorder were standardized according to the publication recommendations by [45] (e.g. type of electrodes, compound for electrodes etc.). Each measurement was started at least five minutes before the beginning of the paradigms to allow for the electrolyte concentration to reach a stable level and was measured through the whole session, start and end of each paradigm was marked automatically by a marker send from Presentation software to Varioport.

#### Measurement and coding of facial expressions

The video stream was digitally recorded on the notebook with 512 kb/s in mp4 format during the whole procedure. A time stamp was included to allow for identification of experimental phases. In a first step a coder trained in Facial Action Coding System (FACS, [46]) unaware of condition or subject group coded action units (AU) 12 (zygomatic major) and 6 (orbicularis occuli), which are basic components of an happy expression. Codings of a happy expression required AU 6 in combination with AU 12. In the FACS system every AU can be coded with a 5 point intensity measure. The timeline was coded online according to intensity and duration of AU 6 and AU 12, as proposed by Ekman and Friesen [46].

### Statistical Analyses

All analyses were performed with SPSS 15 (SPSS Inc., Chicago, USA). To test the effectiveness of mood induction, reactions in the three response systems (experiental/subjective, behavioural/expressive, physiological) were analysed using separate ANOVAs. For the clarification of dysfunctions in the three response systems specific to MDD we conducted a data driven discrimination of the groups.

#### Behavioural data

For analyses of behavioural mood induction effects (PANAS), we relied on the PANAS ratings. The dependent measure for quantifying the mood induction effect was derived from the ratings of positive and negative affect scores of the PANAS, consisting of the sum of 10 positive/negative item ratings divided by 10 ([1], similar to [26], [27]). Repeated measures ANOVAs were calculated for PANAS values (see Table 2 for details). In the analysis of PANAS ratings we included a fourth factor (positive/negative PANAS affective ratings). Both analyses were performed to validate the mood induction effect.

**Table 2:** Mean ESR and PANAS values for patients and controls in the two mood induction conditions.

	Patients in NMI	Controls in NMI	Difference (t and p values)	Patients in HMI	Controls in HMI	Difference (t and p values)	Patients in C	Controls in C	Difference (t and p values)
Anger	1.06 (.25)	1.0 (0)	t_30_ = 1.0 p = .33	1.063 (.25)	1.0 (0)	t_30_ = 1.0 p = 0.325	1.13 (.5)	1.0 (0)	t_30_ = 1.0 p = .325
Disgust	1.0 (0)	1.0 (0)	t_30_ = n.a. p = n.a.	1.0 (0)	1.0 (0)	t_30_ = n.a p = n.a	1.0 (0)	1.0 (0)	t_30_ = n.a p = n.a
Happiness	2.0 (.89)	2.06 (.997)	t_30_ = −0.18 p = .85	2.63 (1.02)	2.75 (.93)	t_30_ = −.36 p = .72	2.38 (.8)	2.6 (1.08)	t_30_ = −.73 p = .466
Sadness	1.19 (.4)	1.06 (.25)	t_30_ = 1.0 p = .3	1.19 (.54)	1.0 (0)	t_30_ = 1.37 p = .178	1.5 (1.03)	1.0 (0)	t_30_ = 1.93 p = .062
Surprise	1.63 (.96)	1.69 (.79)	t_30_ = −.20 p = .842	2.0 (1.03)	1.688 (.87)	t_30_ = .95 p = .363	2.0 (.96)	1.81 (.91)	t_30_ = .56 p = .576
Fear	1.25 (1.0)	1.0 (0)	t_30_ = 1.0 p = .33	1.31 (1.01)	1.063 (.25)	t_30_ = .95 p = .346	1.25 (1.0)	1.0 (0)	t_30_ = 1.0 p = .325
Pos. PANAS	2.38 (.57)	2.27 (.78)	t_30_ = .49 p = .627	2.58 (.62)	2.51 (.76)	t_30_ = .28 p = .782	2.63 (.53)	2.2 (.75)	t_30_ = 1.856 p = .74
Neg. PANAS	1.25 (.29)	1.02 (.05)	**t_30_ = 3.17 p = .003**	1.15 (.22)	1.03 (.05)	**t_30_ = 2.18 p = .037**	1.21 (.28)	1.02 (.44)	**t_30_ = 2.66** **p = .012**

Significant differences are marked in bold. HMI = happy mood induction; NMI = neutral mood induction; C = cartoons and neutral pictures.

#### SCR analyses

For analysis we used Variograph software (BeckerMeditec, Karlsruhe, Germany) and developed an additional MATLAB script (TheMathWorks, Natick, Mass., USA) for detection of skin conductance reactions (SCR). Criterion for a single SCR was a minimum amplitude of 0.02 µSiemens (µS) and a rise time to peak between 0.5 and one second [23]. SCR retained by these criteria were additionally visually inspected to rule out artefacts [23]. Afterwards, data were z-standardized excluding SCRs two standard deviations out of range. Finally, standardized values were averaged for each experimental condition and entered into the analysis.

Differences in peripheral physiological responses were analysed with a three factorial ANOVA, again with Group and Gender as between-subject variables and Mood Induction (HMI, NMI, C) as within-subject factor.

#### Facial expressions

For data reduction purposes and to ensure comparability of the procedures we randomly took 10 frames (from each experimental phase [47]) which were coded by a coder unaware of condition or subject group. Based on intensity of happy facial expressions each frame was given a value between 0 (no signs of happy facial expression) and 10 (highest intensity for AU 12 and AU 6 combined). From these 10 scores a mean intensity score was derived for each condition and participant, which was entered in subsequent analyses.

Differential facial expressiveness in the mood induction procedure and between groups related to activation of AU12 and AU6 were analyzed. We obtained a mean intensity for each mood induction condition as described above and calculated a ANOVA with three factors, again with gender and group as between-subject variables and mood induction as within-subject factor.

#### Data driven discrimination of groups

For determination of variables discriminating between groups we additionally calculated a discriminant analysis including positive and negative self reported affect, SCR and facial expressions in NMI, HMI and C as predictors. As inclusion criterion we took a forward selection based on Wilks Lambda. Critical F value for inclusion was *F* = 3.84 and for exclusion *F* = 2.71, which are default values in SPSS.

## Results

### Behavioural data

**Figure 2:**
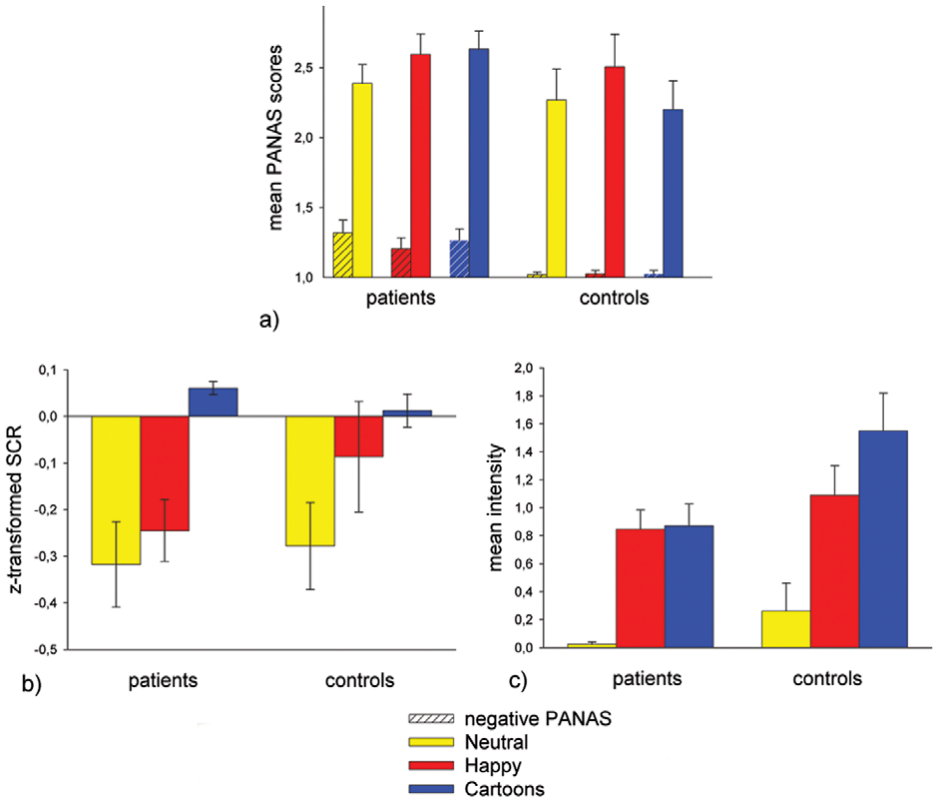
Display of mean values for the subjective, behavioural and physiological variables. a) mean SCR by group and condition b) mean positive and negative PANAS values by group and condition c) mean intensity of happy facial expression by group and condition.

For the PANAS we found a significant main effect of positive/negative PANAS scores, positive and negative affective self ratings differed significantly (*F*(1,29) = 151.624; *p*<0.001, *η_p_^2^* = 0.839), with higher positive than negative affect self ratings. The main effect for condition (e.g. mood induction procedure) was not significant, although a trend can be observed (*F*(2,28) = 2.765; *p* = 0.08; *η_p_^2^* = 0.165). Additionally, we observed a significant interaction between positive/negative PANAS and condition (e.g. mood induction procedure) (*F*(2,28) = 6.68; *p*<0.05, *η_p_^2^* = 0.329). This interaction indicates a successful mood induction with higher positive ratings and lower negative ratings during happy mood in comparison to neutral mood induction in each group (see Fig. 2b). A successful mood induction effect has been shown before by the interaction between positive and negative PANAS values and mood induction procedure. [10], [26], [27], There was only a trend for a main group effect with patients reporting higher negative as well as positive affect after the three experimental conditions (*F*(1,29) = 4.101; *p* = 0.052, *η_p_^2^* = 0.124). T-tests revealed significant group differences only for negative PANAS scores in all three conditions (see Table 2).

Interestingly, the same trend could be observed for gender (*F*(1,29) = 3.227; *p* = 0.083, *η_p_^2^* = 0.1), with male participants expressing both higher positive and higher negative affective ratings.

Nevertheless as the observed trend towards group differences indicates, the non-significance in self ratings between patients and healthy controls probably stems from low power of our sample. Considering the estimated effect size of *η_p_^2^* = 0.124, our model has only a 50% chance of detecting a real significant difference between groups with our sample size.

### SCR

Our analyses showed a main effect for condition (*F*(2,28) = 12.849; *p*<0.001, *η_p_^2^* = 0.479). In a pairwise comparison HMI and NMI did not differ significantly from each other (*p* = 0.063), whereas C differed significantly from the other two conditions (*p*<0.001 for both). Furthermore, a main effect of gender appeared (*F*(1,29) = 4.743; *p*<0.05, *η_p_^2^* = 0.141). Women revealed lower overall SCRs compared to men (see Fig. 2c). Interactions did not reach significance.

### Facial expressions

Here, our analyses demonstrated a main effect of condition (*F*(2,28) = 44.027; *p*<0.001, *η_p_^2^* = 0.759), HMI and C differed significantly from NMI (*p*<0,001), but did not differ significantly from each other. For between subject comparisons we found no significant gender effect. However, as expected, we found a significant group difference (*F*(1,29) = 6.821; *p*<0.001, *η_p_^2^* = 0.190). The most marked difference between groups was in the cartoon condition, with lower mean intensities of happy facial expressions as measured by action units characteristic for happy faces (AU 6 and 12) in the patient group (see Fig. 2d). Interactions did not reach significance.

### Data driven discrimination of groups

The resulting function discriminated well between groups (*R^2^* = 65,3%; *Λ* = 0,574; *p*<0,001). Only negative affect after NMI (*F*(1,30) = 9.639; *p*<0.005), facial expression in C (*F*(2,29) = 7.256; *p*<0.005) and SCR in C (*F*(3,28) = 7.181; *p*<0.005) contributed significantly to discrimination (see Table 3).

**Table 3:** Results of the discriminant analysis.

	F-value	P value	Standardized discriminance coefficient
Negative affect in PANAS rating in neutral mood induction	9.636	0.004	0.742
Mean intensity of facial expressions of happiness in cartoons	7.256	0.003	−0.626
Standardized SCR during cartoons	7.181	0.001	0.639

### Corollary analyses

Patients medicated with SSRI/SNRI (n = 6) did not differ in ratings of psychopathology, self ratings, SCR or facial expressions,(tested with t-tests), from those treated with other medication (n = 10) (for details see Table 1).

BDI scores, age and education as well as TMT-A scores were first entered in all analyses as covariates, but no significant effects were observed and no changes in significance of other variables occurred.

## Discussion

The implementation of two different mood induction procedures drawing on different strategies of mood change, combined with multidimensional assessment enabled us to detect divergent facial and autonomic responses in the patient group. This result is suggestive of an underlying deficit in the patients' ability to adequately express the felt arousal and shows patient's higher emotional physiological reactivity in the cartoon condition. This assumption is underpinned by the discriminant analysis, mainly identifying markers during C to best discriminate between groups of patients and controls, namely SCR and facial expressions during the cartoon presentation. Negative affect after NMI also revealed discriminative power and may reflect an attributional bias. The higher negative affective ratings point to inadequate emotional responses where no emotional response is required. In conclusion, the cartoon condition might be more suitable to uncover deficits in MDD patients experimentally, possibly since the assumed attributional bias is more strongly pronounced in paradigms relying on emotional reactivity in comparison to voluntary, cognitively controlled mood change.

### Comparison of mood induction techniques

In the present study, we applied a well-established positive mood induction procedure based on the presentation of emotional faces to patients with MDD and healthy controls. As found in previous studies with different clinical samples [27] positive mood induction with this procedure was successful, according to subjective ratings. A second mood induction procedure based on the presentation of funny and neutral cartoons, newly developed and standardized by our group, demonstrated largely equivalent and in part even superior effects (in the expressive and physiological domains) in both the patient and the control group, thereby confirming our initial hypotheses of a general effectiveness of these two procedures in altering mood, although both procedures presumably rely on differing strategies. Therefore paradigms seem suitable for future use in neuroimaging studies of mood and emotion in MDD patients and healthy controls.

Furthermore, the multidimensional assessment of emotional responses was able to discern between the two positive mood induction procedures (HMI and C) and the neutral control condition. This indicates the effectiveness of our mood induction procedures with regard to the three response systems of emotional reactivity: (1) experiential/subjective, (2) behavioural/expressive and (3) peripheral physiological responses.

On the experiential level, happiness and positive affective ratings (PANAS) did not show any group differences, thus both the patients and the controls reported corresponding levels of positive affect following the three experimental conditions. We observed significantly higher negative affect (PANAS) in patients in all three conditions. This finding is well in line with previous studies and reflects the negative potentiation view of emotional reactivity (e.g. [3], [5], [19], [33], [48]).

Corresponding to our hypothesis, reduced facial expressivity was observed in the patient group under all three experimental conditions. Specifically, the degree of expressiveness in the patient group was almost identical in the happy mood induction and cartoon condition, whereas the controls displayed more intense facial reactions in cartoons than in the happy mood induction. Studies of facial responses using FACS [49], [50] revealed that experienced exhilaration/amusement induced by jokes or cartoons correlates very highly with smiling and laughter in healthy subjects. Therefore stronger facial expressiveness in the cartoon condition in healthy controls seems to reflect amusement. In contrast, the happy mood induction condition does not provoke this stimulus driven laughter to a similar extent. Cartoons may lead to a strongly externally elicited emotion while the mood induction based on facial expressions is rather a more internally focused method. It represents a more continuous and longer process of remembrance of personal happy events in combination with happy faces. Here, the external facial stimuli are expected to produce contagion. The mood effect may rather represent happiness than amusement. In patients, less frequent smiling following the cartoon condition might reflect the patients' lower cheerfulness and reduced hedonic capacities. Furthermore, reduced modulation of facial expressiveness is common in MDD [51] with some authors emphasizing the fact that facial emotional responsiveness is especially reduced in response to positive stimuli [29]. These findings have also been underpinned by FACS assessment [52]. In particular, patients with depression show reduced facial responsiveness to emotional facial stimuli ([30], [52]–[56]. Low facial expressiveness has previously been classified as motor [57], affective [58] and social impairment [56]. This result indicates a deficit in MDD patients to modulate their expressions according to the context.

With regard to the physiological responses, peripheral physiological activity was related to the properties of the task and stimulus. Both positive mood induction procedures aimed at elevating the current mood, but by completely different means. Skin conductance is known to be related to arousal and strongly reacts to novelty; [23], [24] the mood induction procedure via cartoons confronted our participants with new and potentially arousing emotional stimuli, which might explain elevated skin conductance. Diminished SCRs in the happy mood induction might have been brought about by habituation to the rather invariant facial stimuli, self-paced presentation and the comparatively rather constant and more steady mood state reached by intentional attempts to get into the mood via autobiographical imagination [23], whereas in the cartoon condition, the mood change was much more stimulus driven and largely dependent upon emotional reactivity.

The finding of enhanced negative affect is consistent with the negative potentiation view of emotional reactivity. This view is also linked to the negativity bias proposed in MDD patients, which proposes that MDD patients selectively focus on negative aspects of experiences and exclude positive aspects of experiences [59].

Our results do not match with positive attenuation and the emotion context insensitivity (ECI) view of emotional reactivity in MDD. This view postulates valence-independent deficits in emotional reactivity in MDD [5], with depressed patients displaying stereotyped and inflexible responses to emotional stimuli and a loss of context-appropriate modulation of emotion [33]. Our findings of context-independent enhancement of negative affect, higher autonomic reaction (SCR) and reduced positive facial expressions does not support this hypothesis of context-insensitive processing of emotion in MDD, which would predict blunted emotional reactivity in all three domains regardless of valence.

In terms of Damasio's somatic marker theory the elevated physiological arousal (SCR) in the cartoon condition might be interpreted as over-active somatic markers in MDD patients leading to biased attributional processes (e.g. subjective self ratings) and thus possibly to altered emotional output, i.e. subjective ratings.

### Gender aspects

Gender differences in emotional reactivity have consistently been described. Women have been noted to be more expressive than men [60] although men and women do not differ in self-reports of experienced emotion [61], probably due to stronger susceptibility of expressive behaviour to various social factors [62]. Another hypothesis [63], [64] holds that women tend to be “externalizers” in that their display of emotion tends to be primarily in the expressive domain, whereas men tend to be “internalizers” in that their display of emotion is manifested primarily via the psychophysiological domain. Our results have some bearing on this view, as in our sample both female MDD patients and healthy women revealed lower overall SCRs compared to male MDD patients and healthy men who in turn tended to give higher affective ratings. Further research using larger sample sizes and probably more than only one measure of physiological response (e.g. heart rate, respiratory rate) is needed to reliably assess gender differences, especially with regard to differential gender differences in MDD patients compared to healthy subjects.

### Limitations

While the present study adds to our knowledge about emotional reactivity in MDD, some limitations have to be considered: The assessment of emotional reactivity was conducted in a laboratory setting. Whether this generalizes to naturalistic settings is an open question. Furthermore, the patients were on various antidepressant medication and we were therefore unable to obtain a reliable estimate of the effects of medication on mood and emotional reactivity [65].

Further research is needed to investigate whether our findings can be replicated in unmedicated patients, in patients in remission, and whether they also apply to other mood induction procedures. Low positive affect in MDD interacts with mood-congruent cognitive bias, thereby probably sustaining depressive symptomatology [66]–[68]. Therefore, our paradigms could also be useful in studies addressing the influence of positive mood induction on cognitive appraisal in MDD.

HamD scores in our sample are relatively low, which could confound the findings of our study. Before inclusion into the study every patient was diagnosed based on a standardized interview using the SCID to ensure that he/she were in a depressive episode. Furthermore, we included only in-patients, i.e. the patients were treated for an acute episode. However, most patients were included into the study in a stabilized psychopathological status to be able to perform this study. Correspondingly, the mean HamD scores are still within the range of mild depressive symptoms. Nevertheless, even facing a mostly remitted depressive symptomatology we are able to demonstrate group differences pointing to stable trait like deficits in emotion processing present even after remission of the pure depressive symptoms.

The applied paradigms are designed for use in neuroimaging studies. Since neuroimaging studies are limited in various ways in comparison to behavioural studies, the paradigms are adjusted to work under these conditions, which might decrease their power to induce the specific emotions, but nevertheless, these procedures have been shown to be effective, both in our sample and in other samples.

### Conclusion

Our results indicate that both procedures are effective for the induction of positive mood states. The multidimensional assessment of emotional reactivity pursued here is superior to single-level assessment. Especially concerning deficits in MDD patients, all three response systems contribute significantly to discrimination of patients and controls. However, relying on self-reported happiness or positive affect alone would not allow reliably determining dysfunctions in patients. Patients showed lower facial expressiveness and higher SCRs in the cartoon condition and negative self-reported affect in all three conditions, which is assumed to indicate a general attributional bias. This attributional bias is most pronounced in the cartoon condition, indicating its advantage over happy mood induction via faces and memory processes to tap into MDD patient's deficits. The disturbed emotional reactivity in patients may exert a stronger influence in the cartoon condition. Dysfunctions in patients are also discussed in terms of different emotion theories, such as the somatic marker theory proposed by Damasio [22] and the negative potentiation view of emotional reactivity, which relates to the negativity bias in depression.

## Supporting Information

Supplementary Material S1 Description of the Cartoon stimulus evaluation and standardization [39].
(DOC)
