# Supplementary material for: Mood Induction in Depressive Patients: A Comparative Multidimensional Approach
Source: PLoS One. 2012 Jan 9;7(1):e30016. doi: 10.1371/journal.pone.0030016 (PMC3253810; doi:10.1371/journal.pone.0030016)
Supplement: Supplementary Material S1 — Description of the Cartoon stimulus evaluation and standardization [39]. (DOC) [file pone.0030016.s001.doc]

*S1: Cartoon stimulus evaluation/standardization*

Cartoon stimuli were evaluated in a prior, preliminary study with 50 different healthy participants (mean age=30, *SD*=10; 26 males), in which we included 205 pictures. All participants had to rate each picture for funniness, perceptibility and valence on a 9 point rating scale (1=not funny at all and 9=very funny). Perceptibility referred to whether the drawings were easily perceivable, the caption readable etc. Valence related to how positive or negative the cartoon was rated, since a joke might be perceived as funny and negative (e.g. morally unsound, [38]) Only pictures with a mean perceptibility rating of above 5 were retained. Cartoons with a mean funniness below 5 and neutral pictures with a mean funniness above 3 were eliminated. The final set included 80 cartoons with a mean funniness rating of 5.5 (*SD*=0.6) and 80 neural pictures with a mean funniness rating of 2.4 (*SD*=0.4). A t-test revealed a significant difference for funniness between neutral pictures and cartoons (*t*(158)= 36.799, *p*<0.001).

In a second step we confirmed our classification by cluster analysis, which classified all cartoons and neutral pictures correctly, an ANOVA of the cluster centres indicated a significant difference (*F*(1,158)=856,851; *p*<0,001).

In a third step we evaluated how much funniness, perceptibility and valence contributed to the classification into the two clusters, therefore we calculated a discriminant analysis with forward selection according to Wilks Lamba. Critical F value for inclusion was *F*=3.84 and for exclusion *F*=2.71, which are default values in SPSS. The predictors consisted of the mean values for the three ratings of each picture and cartoon vs. neutral picture were included as group variables. The three variables discriminated successfully between groups (*R^2^*=92.5%; *Λ*=0,145; *p*<0,001). The funniness rating revealed the strongest contribution to the classification followed by the valence rating and finally the perceptibility rating as demonstrated by the standardized canonical coefficients. Cartoons and neutral pictures were presented self-paced and could be ended by button press (mean=5.5s, *SD*=0.96s). The paradigm has additionally been validated in a fMRI study and shown to lead to differential brain activation between neutral pictures and cartoons [39].
